# A retrospective clinical audit on depression management in line with NICE NG222 guidelines at a primary care setting in rural England

**DOI:** 10.1192/j.eurpsy.2026.10588

**Published:** 2026-07-31

**Authors:** S. U. Rajah, L. Mahesh Kumar

**Affiliations:** School of Medicine and Dentistry, University of Lancashire, Preston, United Kingdom

## Abstract

**Introduction:**

Depression is a globally prevalent mental health condition and a leading cause of disability, frequently managed within UK primary care. The National Institute for Health and Care Excellence (NICE) NG 222 provides guidance for diagnosing and managing depression. This audit is underscored by the discontinuation of national supervision for depression care, a scarcity of audits on the updated guidelines, and the need to assess adherence in a rural setting where patients may face unique barriers to accessing healthcare.

**Objectives:**

To assess and improve the adherence to NICE guideline NG222 “Depression in adults: treatment and management” by auditing the management of newly diagnosed depression, focusing on the documentation of suicide risk assessment and first-line treatment for less-severe depression, therefore to improve patient outcomes.

**Methods:**

A retrospective audit was conducted at a general practice (GP) in rural England. Patients newly diagnosed with depression between June 2022 and May 2025 were identified using an Egton Medical Information Systems (EMIS) search. For the first criterion, a random sample of 20 patients was reviewed for documented suicidal ideation enquiry. For the second, 11 patients with less-severe depression (PHQ-9<16) were assessed to determine if antidepressants were offered as a first-line treatment without documented patient preference. The data extracted was compared to set standards of 95% and 90% respectively.

**Results:**

The practice did not meet the set standards (Table 1). For criterion 1, 85% (17/20 cases) had documented enquiry of suicidal ideation, falling short of the 95% standard (Figure 1). For criterion 2, adherence was 81.8% (9/11 cases), below the 90% standard for offering non-pharmacological treatment first for less-severe depression (Figure 2).

**Table 1.** Results Table 1. Long description.

Criteria	Set Standard (%)	Practice Compliance (%)
Documented enquiry of suicidal intentions/ideations at assessment	95%	85%
Antidepressants not routinely offered first-line for less-severe depression (unless documented patient preference)	90%	81.8%

**Image 1:**
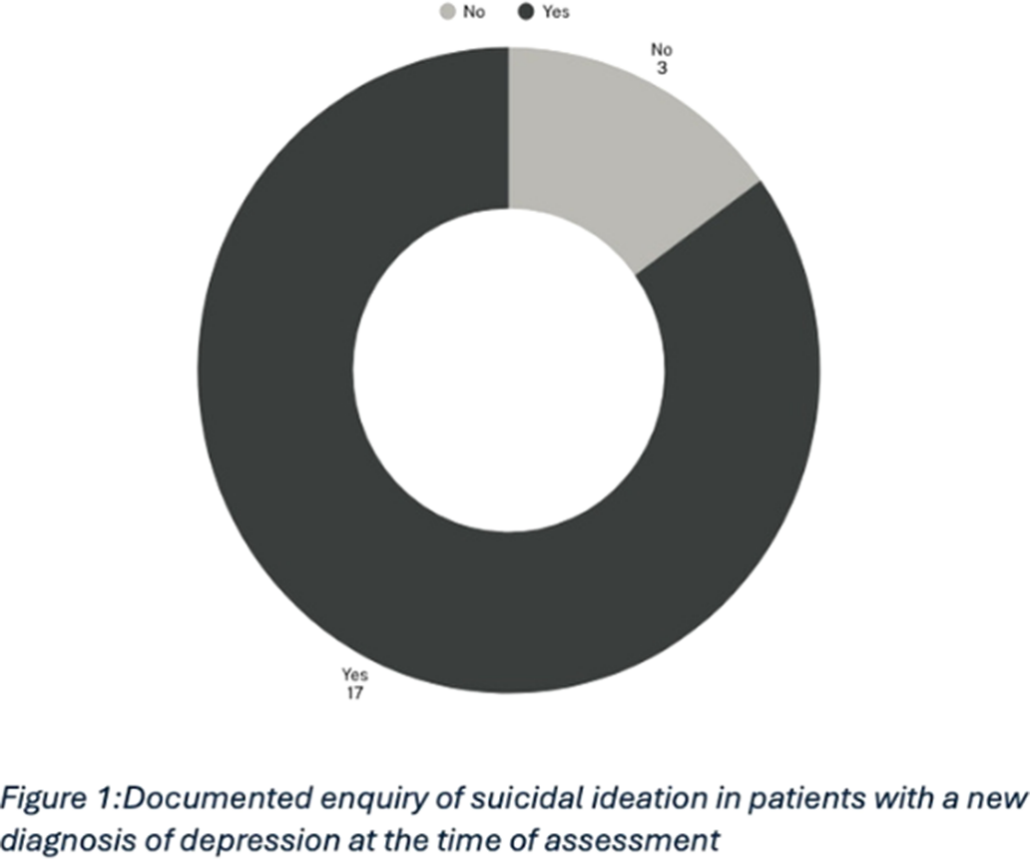

**Image 2:**
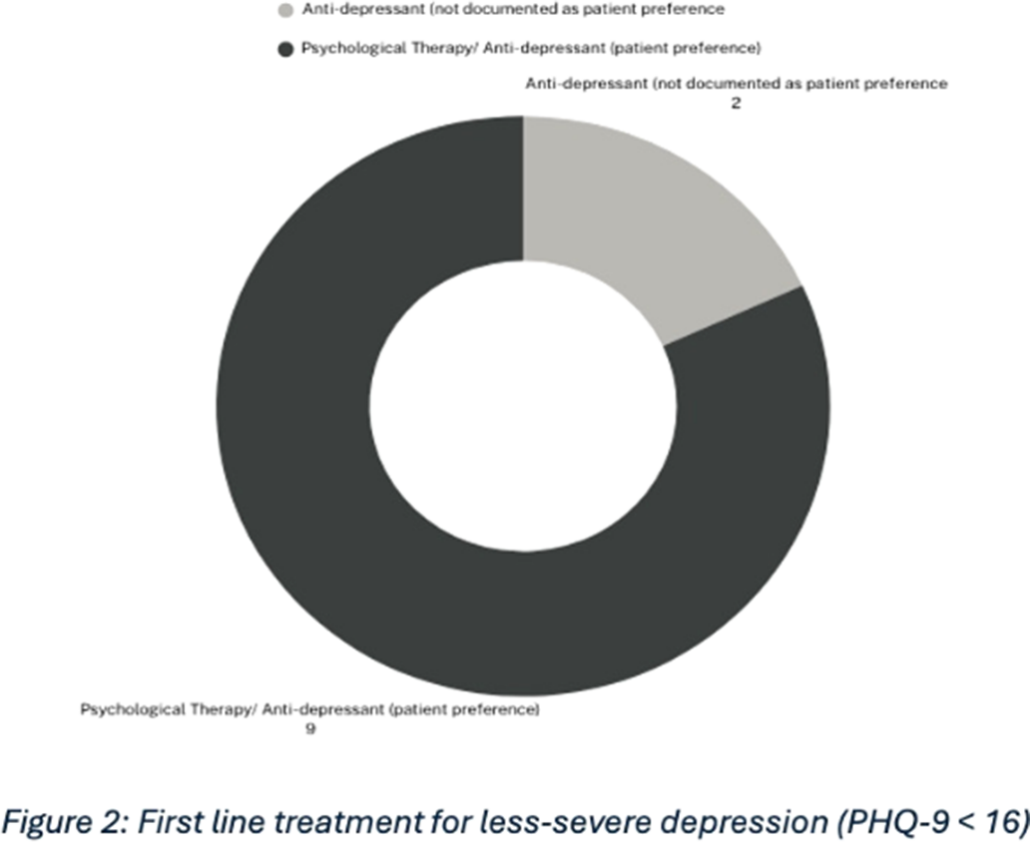

**Conclusions:**

This audit reveals that adherence to NICE NG222 guidelines for depression management was suboptimal, highlighting a significant gap between guidance and practice. Contributing factors may include challenges in remote consultations, inconsistent documentation, and long waiting times for psychological services. While the study’s findings are limited by a small sample size from a single practice and a retrospective design, the identified pitfalls point to areas for improvement. To address this, targeted interventions were proposed: implementing mandatory EMIS prompts and templates to standardise documentation, alongside specific staff training on guideline adherence and shared decision-making. These practical steps aim to improve care and patient safety, with a re-audit planned in 12 months to evaluate their impact.

**Disclosure of Interest:**

None Declared

## Long descriptions

### Table 1. Long description

The table consists of three columns: Criteria, Set Standard (%), and Practice Compliance (%).

* Row 1: Documented enquiry of suicidal intentions/ideations at assessment. Set Standard is 95%. Practice Compliance is 85%.

* Row 2: Antidepressants not routinely offered first-line for less-severe depression (unless documented patient preference). Set Standard is 90%. Practice Compliance is 81.8%.

Navigate back to Table 1..

